# High-Throughput Discovery of Inhibitors Targeting Monkeypox Virus H1 Phosphatase

**DOI:** 10.3390/v17111493

**Published:** 2025-11-12

**Authors:** Chengcheng Tao, Mailikezhati Alifu, Haojun Huang, Zhi Luo, Yaxian Li, Xuecen Guan, Mengmeng Liu, Junchi Hu, Wen Cui, Wei Wang

**Affiliations:** 1 College of Pharmacy, Chongqing Medical University, Chongqing 400016, China; chengchengtao@cqmu.edu.cn (C.T.); 19823008455@163.com (M.A.); haojunhuang@swmu.edu.cn (H.H.); 13310267951@163.com (X.G.); 103000@cqmu.edu.cn (M.L.); 2 Department of Laboratory Medicine, The Affiliated Hospital of Southwest Medical University, Sichuan Province Engineering Technology Research Center of Molecular Diagnosis of Clinical Diseases, Molecular Diagnosis of Clinical Diseases Key Laboratory of Luzhou, Sichuan 646000, China; 3 Center for Novel Target and Therapeutic Intervention, College of Pharmacy, Chongqing Medical University, Chongqing 400016, China; luozhi9797@163.com (Z.L.); liyaxian0829@163.com (Y.L.)

**Keywords:** monkeypox virus, phosphatase H1, enzymatic assay, high-throughput screening, inhibitor, molecular docking analysis

## Abstract

Mpox, caused by monkeypox virus (MPXV), remains a Public Health Emergency of International Concern (PHEIC) and poses a serious global health threat. Current therapeutic options for MPXV infection are limited. The orthopoxvirus dual-specificity phosphatase H1 plays critical roles in suppressing interferon signaling, regulating early viral transcription, and modulating viral core protease activity. Suppressing H1 expression markedly reduces the production of infectious viral particles, highlighting it as a promising antiviral target. Here, we developed a high-throughput enzymatic assay using p-nitrophenyl phosphate as a substrate to discover MPXV H1 inhibitors. We demonstrated that both the N-terminal helix α1, which mediates H1 dimerization, and the catalytic residue Cys110 are indispensable for enzymatic activity, validating them as potential “hot spots” for drug design. Screening identified 17 potent inhibitors with nanomolar IC_50_ values and minimal cytotoxicity. Molecular docking revealed that these inhibitors bind within the active site of MPXV H1, interacting with residues in the P-loop and WPD-loop, thereby restricting substrate access and suppressing activity. This study identifies several potent inhibitors of MPXV H1, establishing a foundation for the development of novel antivirals against MPXV infection.

## 1. Introduction

Mpox, caused by monkeypox virus (MPXV), has spread globally since May 2022. Over the past three years, the World Health Organization (WHO) has declared two Public Health Emergencies of International Concern (PHEIC) related to mpox. To date, more than 150,000 confirmed cases and 380 deaths have been reported. Antivirals initially developed for smallpox, including tecovirimat, brincidofovir, and cidofovir, have been evaluated against MPXV infection [1]. However, a recent clinical trial demonstrated that tecovirimat did not shorten mpox symptom duration compared with placebo [2]. While brincidofovir and cidofovir show antiviral activity in cell and animal studies, their clinical use is constrained by safety concerns [3,4]. No antivirals have demonstrated efficacy against MPXV infection in controlled clinical trials, underscoring the urgent need for new therapeutic candidates.

MPXV is a member of the Orthopoxvirus genus, which also includes vaccinia virus (VACV), cowpox virus (CPXV), and variola virus (VARV) [5,6]. MPXV possess linear double-stranded DNA (dsDNA) genomes of ~200 kb encoding ~190 open reading frames essential for viral replication, pathogenesis, and immune evasion [7,8]. Upon entry, specialized lateral bodies deliver viral effector proteins into the host cytosol, including the dual-specificity phosphatase H1 [9]. Suppressing H1 markedly reduces the production of infectious viral particles [10,11]. Mechanistic studies reveal that H1 regulates phosphorylation of the viral early transcription factor A7, whose hyperphosphorylation lead to transcription deficiency [11,12]. H1 also modulates host immunity by dephosphorylating STAT1, thereby blocking interferon-γ (IFN-γ) signaling [13,14]. More recently, H1 has been implicated in virion maturation through regulation of the viral core protease (Core^Pro^); H1-deficient virions harbor hyperphosphorylated Core^Pro^ at S134, leading to aberrant substrate cleavage [12]. Thus, H1 functions at the interface of viral replication and immune evasion, making it an attractive target for antiviral development.

We previously reported a 1.8-Å crystal structure of MPXV H1, which revealed a butterfly-like, domain-swapped dimer. The N-terminal helix α1 is exchanged between monomers, forming a stable dimer interface critical for function [15]. This dimerization mode is conserved across poxvirus H1 proteins, and disruption of α1-mediated dimerization impairs dephosphorylation of STAT1 [13]. The two catalytic sites are located ~39 Å apart on the same side of the dimer, each comprising a Cys-Arg-Asp triad essential for dephosphorylation [13,15]. These structural insights identify two promising “hot spots” for inhibitor design: the dimer interface and the catalytic site. However, no systematic high-throughput screening for MPXV H1 inhibitors has been reported.

In this study, we established a high-throughput enzymatic assay using p-nitrophenyl phosphate (pNPP) as the substrate. The wild-type MPXV H1 exhibited robust catalytic activity with a Michaelis constant (*K*_m_) of 1.5 mM toward pNPP. Functional analyses confirmed that deletion of helix α1 or substitution of Cys110 with serine significantly impaired enzymatic activity. Screening the L4000 compound library (7065 small molecules) yielded 76 compounds with significant H1 inhibitory activity. Subsequent validation identified 17 candidates with potent inhibition, nanomolar-range IC_50_ values, and minimal cytotoxicity. Molecular docking revealed that five inhibitors occupy the substrate-binding site, suggesting direct competition with substrate access and inhibition of dephosphorylation. Together, our findings identify potent inhibitors of MPXV H1, providing critical leads for the development of novel antiviral agents against MPXV infection.

## 2. Materials and Methods

### 2.1. Cloning, Expression, and Purification of MPXV H1 and Variants

The wild-type (WT) MPXV H1 coding sequence (GenBank: ON563414) was codon-optimized for *Escherichia coli* (*E. coli*) expression and commercially synthesized (Tsingke Biotech, Beijing, China). The optimized sequence was cloned into the pETDuet-1 vector via *EcoR* I and *Nde* I restriction sites. Site-directed mutagenesis was carried out using a Mut Express II Fast Mutagenesis Kit V2 (Vazyme Biotech, Nanjing, China), and all constructs were verified by sequencing.

For protein expression, *E. coli* BL21 (DE3) cells were transformed with the respective plasmids. Expression was induced with 1 mM isopropyl β-D-1-thiogalactopyranoside (IPTG) when the optical density at 600 nm (OD_600_) reached 0.6–0.8. Cultures were incubated at 16 °C for 16 h, after which cells were harvested by centrifugation. Pellets were resuspended in lysis buffer (20 mM Tris-HCl, pH 8.0, 500 mM NaCl, 10 mM imidazole, 5 mM MgCl_2_, 10% glycerol) and lysed using a high-pressure homogenizer. Insoluble debris was removed by centrifugation (16,000× *g*). The clarified lysate was subjected to Ni-NTA affinity chromatography (Cytiva) at 4 °C. Columns were washed with ≥10 column volumes (CV) of wash buffer (20 mM Tris-HCl, pH 8.0, 200 mM NaCl, 50 mM imidazole, 5 mM MgCl_2_, 10% glycerol), and proteins were eluted with buffer containing 250 mM imidazole. Fractions were further purified by heparin affinity chromatography (HiTrap Heparin HP, Cytiva, Marlborough, MA, USA) and size-exclusion chromatography (SEC) using a Superdex 200 Increase 10/300 GL column. Proteins were stored in SEC buffer (20 mM Tris-HCl, pH 8.0, 500 mM NaCl, 5 mM MgCl_2_).

### 2.2. Dephosphorylation Activity Assay

MPXV H1 activity was assayed using p-nitrophenyl phosphate (pNPP) as the substrate [16]. Hydrolysis of pNPP generates p-nitrophenol (pNP), which was quantified spectrophotometrically at 405 nm.

To determine kinetic parameters, reactions containing 0.24 μM MPXV H1 and pNPP concentrations ranging from 0.05–5 mM were initiated by adding enzyme to pre-warmed substrate in assay buffer (100 mM Bis-Tris pH 6.5, 1 mM DTT, 1 mM EDTA, 10% DMSO) at 37 °C. Absorbance at 405 nm was recorded every 30 s for 3 min using a SpectraMax iD5 microplate reader (Molecular Devices). pNP concentrations were calculated using the molar extinction coefficient (ε405 = 18,000 M^−1^cm^−1^). Initial velocities (*V*_0_) were derived from the linear range of product formation curves, and kinetic constants (*K*_m_ and *V*_max_) were obtained by fitting to the Michaelis–Menten equation (GraphPad Prism 8).

Relative activities of MPXV H1 variants were compared by incubating 1.2 μM of each variant with 1 mM pNPP for 30 min at 37 °C, followed by absorbance measurement at 405 nm. Enzyme-free reactions served as negative controls.

The inhibitory activity of protein tyrosine phosphatase (PTP) inhibitor IV was assessed using 1.2 μM enzyme and 1.5 mM pNPP with inhibitor concentrations ranging from 1.5–400 μM. MPXV H1 was preincubated with the inhibitor at room temperature (RT) for 12 h before initiating reactions at 37 °C. Inhibitory efficacy was calculated as:

Inhibitoryefficacy = 1−Ratewith inhibitorRatewithout inhibitor×100%

The IC_50_ value was determined by fitting dose–response data to a sigmoidal equation using GraphPad Prism.

### 2.3. High-Throughput Inhibitor Screening

High-throughput screening was performed using the Explore G3 integrated workstation and the L4000 bioactive compound library (TargetMol, Boston, MA, USA), which contains 7065 compounds. In each reaction, 1.2 μM MPXV H1 was incubated with 25 μM test compound in 384-well plates at RT for 12 h. The reaction was initiated with pNPP (final concentration 1.5 mM), and pNP production was quantified after 3 min using an EnSight multimode plate reader. PTP inhibitor IV served as the positive control, and reactions without inhibitors served as the negative control. Compounds showing >95% inhibition in the first screen were subjected to a second validation screen.

### 2.4. IC_50_ Determination

IC_50_ values of candidate compounds were determined as described for PTP inhibitor IV. In the first round, reactions contained 1.2 μM enzyme, 1.5 mM pNPP, and inhibitor concentrations ranging from 97.7 nM–25 μM. A second round with refined concentration ranges was conducted based on initial results. All assays were performed in triplicate, and results are reported as mean ± standard deviation.

### 2.5. Cell Viability Assay

Cytotoxicity of candidate inhibitors was evaluated using a Cell Counting Kit-8 (CCK-8; Beyotime Biotechnology, Shanghai, China), which uses a compound named water-soluble tetrazolium 8 (WST-8) for evaluation of cell viability. HeLa cells were seeded at 1.0 × 10^4^ cells/well in 96-well plates with 100 μL complete medium containing 10 μM test compound. DMSO (0.1% *v*/*v*) served as the negative control. After 24 h of incubation at 37 °C, 10 μL CCK-8 reagent was added to each well and incubated for 2 h. Absorbance was measured at 450 nm with a microplate reader.

### 2.6. Molecular Docking

Molecular docking was performed using the CDOCKER module in Discovery Studio, followed by energy minimization using the CHARMm force field. Compound structures were retrieved from PubChem (https://pubchem.ncbi.nlm.nih.gov/) (accessed on 20 March 2025). The MPXV H1 structure (PDB ID: 8GZ4; 1.80 Å resolution) was prepared using the Prepare Protein tool, defining catalytic residues Asp79, Cys110, and Arg116 as the docking site. Representative compounds were docked, and binding modes were visualized using PyMOL 2.0.7 (Schrödinger, LLC, New York, NY, USA).

## 3. Results

### 3.1. Development of an Enzymatic Assay for MPXV H1 Activity

**Figure 1:**
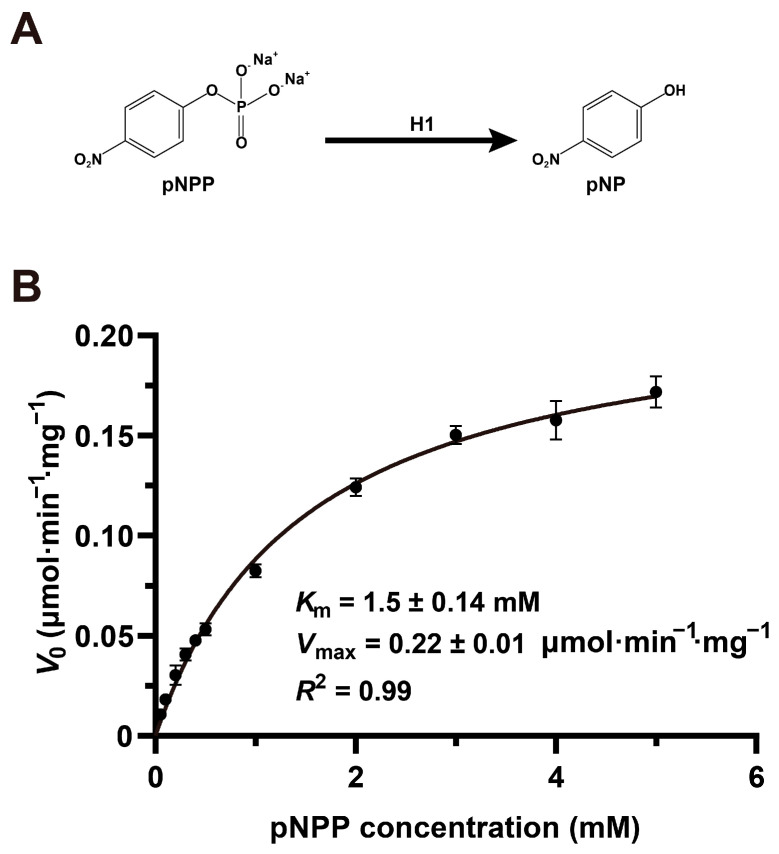
Enzymatic activity assay and Michaelis-Menten kinetic characterization of MPXV H1. (**A**) The dephosphorylation reaction catalyzed by MPXV H1 was monitored by quantifying pNP production via absorbance measurements at 405 nm. (**B**) Michaelis-Menten kinetic characterization of MPXV H1 using a range of pNPP concentrations. *K*_m_, Michaelis constant; *V*_max_, maximum velocity; *V*_0_, initial reaction velocity; *R*^2^, coefficient of determination. All assays were performed in triplicate, with mean values and standard deviations plotted.

To enable high-throughput inhibitor screening, we established an enzymatic assay for MPXV H1. The enzyme catalyzes the hydrolysis of pNPP, producing pNP and inorganic phosphate. Because pNP exhibits a strong absorbance peak at 405 nm, its accumulation served as a direct readout of enzymatic activity (Figure 1A). Michaelis–Menten kinetics were determined across a pNPP concentration range of 0.05–5 mM. MPXV H1 displayed typical saturation kinetics, with a maximum velocity (*V*_max_) of 0.22 μmol·min^−1^·mg^−1^ and a Michaelis constant (*K*_m_) of 1.5 mM, confirming that the wild-type (WT) enzyme is catalytically active under these conditions.

### 3.2. Validation of the Dimer Interface and Catalytic Site as Inhibitor “Hot Spots”

Structural analysis of MPXV H1 identified two regions as potential druggable “hot spots”: the dimer interface and the catalytic pocket. To validate their functional importance, we generated two variants: one that disrupts dimerization and another that targets the catalytic residue.

**Figure 2:**
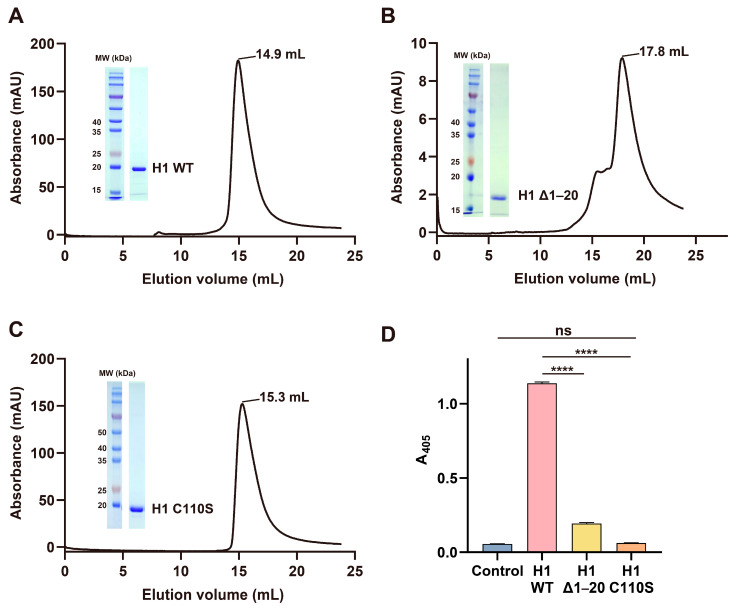
Size-exclusion chromatography and relative activity of MPXV H1 variants. Size-exclusion chromatography (SEC) analysis and SDS-PAGE of (**A**) WT MPXV H1, (**B**) MPXV H1 Δ1–20, and (**C**) MPXV H1 C110S. (**D**) Comparison of enzymatic activities of MPXV H1 variants. A control reaction without enzyme was included. All activity assays were performed in triplicate, with mean values and standard deviations plotted. ns, not significant. **** *p* < 0.0001.

To assess the role of helix α1 in dimerization, we constructed a deletion mutant lacking residues 1–20 (Δ1–20). Size-exclusion chromatography (SEC) revealed that WT H1 eluted at 14.9 mL, corresponding to a ~40 kDa dimer (Figure 2A), whereas Δ1–20 eluted at 17.8 mL, consistent with a ~20 kDa monomer (Figure 2B). Thus, deletion of helix α1 effectively disrupted dimerization. Functionally, WT H1 showed strong activity: a 30-min reaction with 1.2 μM enzyme and 1.5 mM pNPP produced an absorbance (A405) of 1.14, indicating robust pNP generation. In contrast, Δ1–20 displayed markedly reduced activity, with A405 reaching only 0.19, marginally above the negative control (Figure 2D). These results demonstrate that helix α1 is essential for both dimer formation and enzymatic activity.

We next examined the role of the catalytic residue Cys110. This residue directly attacks the phosphorus atom during the dephosphorylation reaction, generating a transient enzyme-phosphate intermediate. The intermediate is then hydrolyzed to release inorganic phosphate and regenerate the enzyme. The C110S substitution yielded a protein with elution behavior comparable to WT H1 (15.3 mL; Figure 2C) but completely abolished catalytic activity (Figure 2D). This confirms the indispensability of C110 for catalysis and highlights the active site as a key inhibitory target.

### 3.3. Protein Tyrosine Phosphatase Inhibitor IV Potently Inhibits MPXV H1

**Figure 3:**
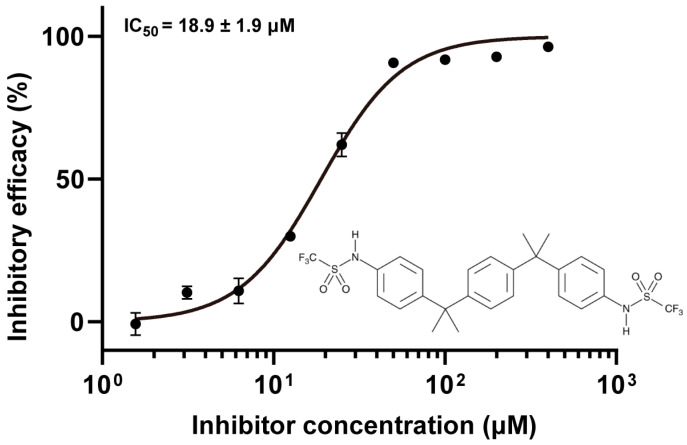
Inhibitory activity of PTP inhibitor IV against MPXV H1. The inhibitory efficacy of PTP inhibitor IV was assessed across concentrations ranging from 1.5 to 400 μM, and the resulting data were fitted to determine the IC_50_ value. The inhibition assays were conducted in triplicate, with mean values and standard deviations plotted.

A positive control inhibitor was required for subsequent screening. Protein tyrosine phosphatase (PTP) inhibitor IV is known to target the catalytic triad of PTPs and effectively inhibits VARV H1 with low-micromolar potency [17,18]. Because this catalytic triad is conserved across poxvirus H1 homologs, we tested its efficacy against MPXV H1. Consistent with expectations, PTP inhibitor IV strongly inhibited MPXV H1, yielding an IC_50_ of 18.9 μM (Figure 3). It was therefore adopted as the positive control in high-throughput screening experiments.

### 3.4. High-Throughput Screening of MPXV H1 Inhibitors

**Figure 4:**
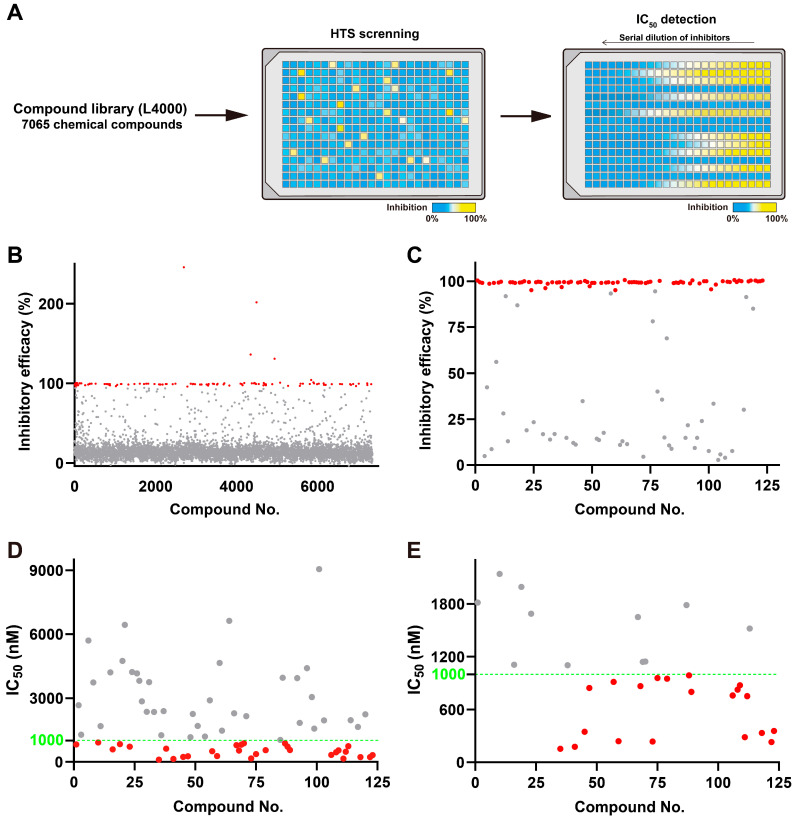
High-throughput screening of inhibitors targeting MPXV H1 and IC_50_ determination. (**A**) Schematic illustration of the high-throughput inhibitor screening process. (**B**) The first round of high-throughput screening identified 123 candidate compounds. (**C**) A subsequent round of screening confirmed the inhibitory activities of 76 compounds. Compounds exhibiting inhibitory efficacy greater than 95% are indicated by red dots, whereas those with lower efficacy are shown in gray. (**D**,**E**) IC_50_ determination of the candidate compounds. The green dashed line marks the IC_50_ threshold of 1000 nM; red dots represent compounds with IC_50_ values below this threshold, while gray dots denote those above it.

To identify novel inhibitors of MPXV H1, we performed high-throughput screening of the L4000 bioactive compound library, which contains 7065 chemical compounds (Figure 4A). Screening was conducted with 1.2 μM MPXV H1 and 25 μM of each compound. At this concentration, PTP inhibitor IV completely inhibited MPXV H1 activity, validating the assay setup. The first-round screen identified 123 compounds with >95% inhibition. These were renumbered as compounds **1**–**123** and retested under identical conditions (Figure 4B). Of these, 76 compounds consistently retained >95% inhibition (Figure 4C) and were selected for IC_50_ determination.

Dose–response analysis revealed that 31 of the 76 compounds exhibited IC_50_ values below 1 μM (Figure 4D and Figure S1). These candidates were re-evaluated under optimized concentration ranges, yielding 20 compounds that consistently displayed sub-micromolar IC_50_ values (Figure 4E and Figure S2). A summary of these top candidates is provided in Table 1.

To further assess therapeutic potential, we tested the cytotoxicity of the 20 compounds using a CCK-8 viability assay in HeLa cells. Cells were treated with 10 μM compound for 24 h, followed by absorbance-based viability measurements (Figure S3). Seventeen compounds maintained >85% cell viability, with compounds No. **41**, **45**, **47**, **57**, **88**, **89**, **109**, and **123** exhibiting particularly low cytotoxicity. In contrast, compounds No. **68**, **75**, and **108** induced substantial cytotoxicity. These 17 compounds, therefore, represent promising candidates for further investigation.

### 3.5. Inhibitory Mechanism of Candidate Compounds

To elucidate the mechanisms by which candidate compounds inhibit MPXV H1, we first attempted to obtain high-resolution crystal structures of H1–inhibitor complexes. Although both soaking and co-crystallization trials yielded crystals, no interpretable electron density corresponding to the inhibitors was observed in the refined structures. We therefore employed molecular docking to investigate potential binding modes within the active site.

**Figure 5:**
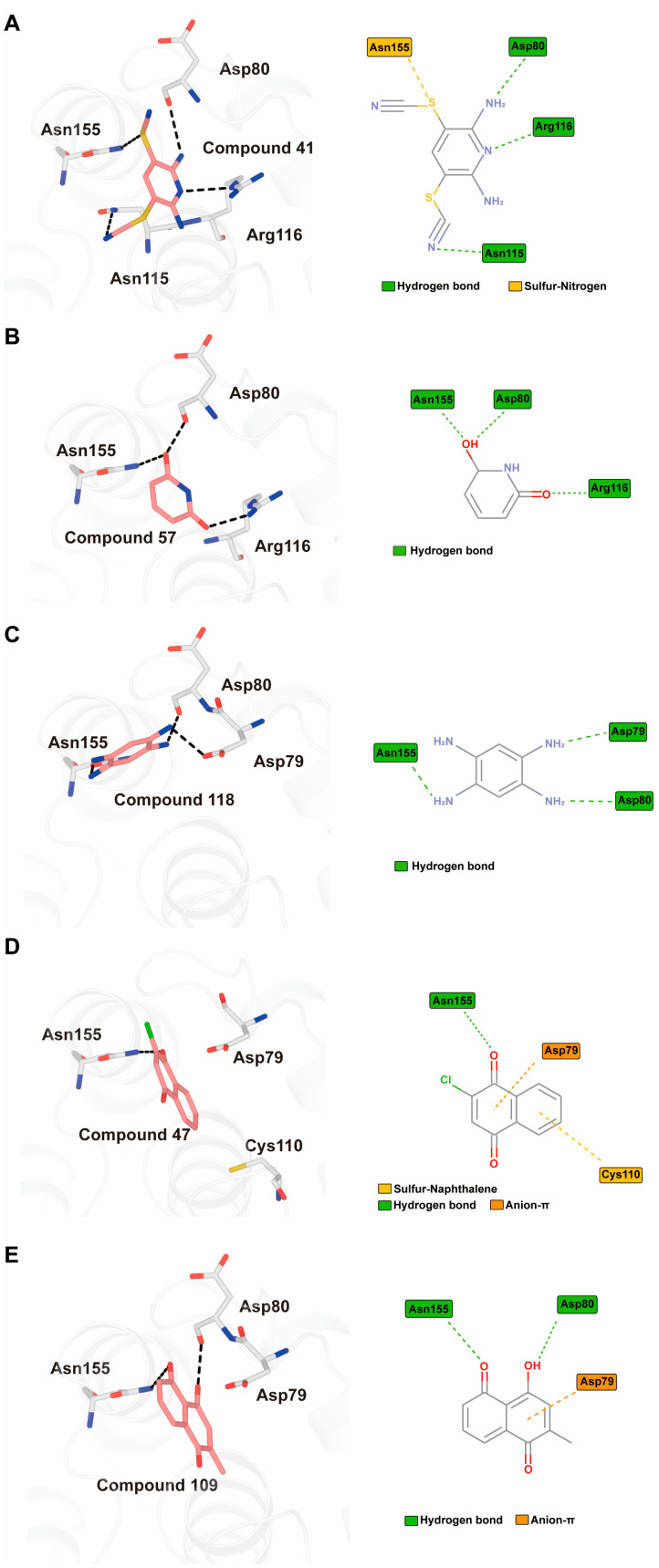
Interactions between representative inhibitors and MPXV H1. The interactions between MPXV H1 and compounds (**A**) No. **41**, (**B**) No. **57**, (**C**) No. **118**, (**D**) No. **47** and (**E**) No. **109** are shown. The compounds and the residues involved in the interaction are shown as sticks in the structural models. Hydrogen bonds are shown as black dashed lines. The 2D diagrams highlighting the types of interactions are shown to the right of the structural models.

Using the CDOCKER module in Discovery Studio, five of the seventeen candidate compounds were successfully docked into the H1 catalytic pocket, all of which comprise one or two rings. The calculated CDOCKER energy values, reflecting receptor–ligand interactions and internal strain energies, are summarized in Table 2. Among these, compound No. **41** (PR619), one of the most potent inhibitors, showed the lowest CDOCKER energy of −24.9 kcal/mol, consistent with strong binding affinity. Docking revealed that PR619 establishes hydrogen bonds with Arg116, Asn115, and Asp80, while the thiocyanate sulfur at the 5-position engages in a sulfur–nitrogen interaction with Asn155 (Figure 5A) [19]. These interactions likely underlie both the favorable docking energy and its potent inhibitory activity.

Compound No. **57** (2,6-dihydroxypyridine hydrochloride), which shares a central pyridine scaffold with PR619, forms hydrogen bonds via its hydroxyl groups with Asn155, Asp80, and Arg116. However, lacking the thiocyanate moiety present in PR619, it displayed a higher (less favorable) CDOCKER energy of −13.2 kcal/mol and reduced potency, highlighting the contribution of the thiocyanate-mediated interactions (Figure 5B). Compound No. 118 (1,2,4,5-benzenetetramine tetrahydrochloride) replaces the pyridine ring with a benzene core bearing four amine substituents. Its amine groups form hydrogen bonds with Asp79, Asp80, and Asn155, yielding a docking energy of −22.5 kcal/mol, comparable to PR619 and indicative of stable binding (Figure 5C).

Compound No. **47** (2-chloro-1,4-naphthoquinone) displayed a docking energy of −16 kcal/mol. The carbonyl group at the 1-position forms a hydrogen bond with Asn155, while the naphthalene ring engages in an anion–π interaction with Asp79 and a sulfur–naphthalene interaction with Cys110 (Figure 5D) [20]. Compound No. **109** (Plumbagin), a structural analog of compound **47**, differs by carrying a methyl group at the 2-position in place of chlorine, along with a hydroxyl substituent at the 5-position. Docking results indicated hydrogen bonds between the 5-ketone oxygen and Asn155, and between the 4-hydroxyl group and Asp80. The naphthalene ring additionally participates in an anion–π interaction with Asp79, closely mirroring the binding mode of compound **47** (Figure 5E).

Taken together, the five inhibitors share a conjugated aromatic core, typically comprising one or two six-membered rings decorated with polar oxygen- or nitrogen-containing substituents. All dock within the catalytic pocket, forming non-covalent interactions predominantly with hydrophilic residues, including Asp79, Asp80, Asn115, Asn155, and Arg116. These binding features suggest direct competition with substrate binding. The docking analyses thus provide mechanistic insights into inhibitor engagement with MPXV H1 and may guide future optimization of these compounds.

## 4. Discussion

The dual-specificity phosphatase H1 of MPXV plays a central role in viral replication by dephosphorylating both phosphotyrosine and phosphoserine/threonine residues and has thus emerged as a promising antiviral target [21]. In this study, we characterized MPXV H1 activity and demonstrated that both the N-terminal helix α1 and the catalytic residue Cys110 are indispensable for enzymatic function, validating them as potential inhibitory “hot spots.” Through high-throughput screening (HTS), we identified 17 compounds with potent activity (IC_50_ < 1 µM) and minimal cytotoxicity. Docking analyses further elucidated their binding modes within the catalytic pocket. Together, these findings provide mechanistic insight into MPXV H1-mediated dephosphorylation and establish a foundation for developing novel inhibitors targeting this enzyme.

The importance of helix α1 in MPXV H1 function is consistent with prior work showing that disruption of VACV H1 dimerization impairs its ability to dephosphorylate 3-O-methylfluorescein phosphate and phosphorylated STAT1 [13]. Structural studies indicate that helix α1 is located far from the active site [13,15], raising the mechanistic question of how it modulates catalytic activity. One possibility is that dimerization positions the α1 helix of one protomer to induce local conformational changes in the partner, compacting the active site and enhancing catalysis [13,22]. Definitive confirmation will require structural characterization of an H1 variant lacking helix α1. Taken together with previous reports, our results highlight helix α1 as a critical determinant of catalytic activity and suggest that the dimerization interface represents a unique druggable site in poxvirus H1 phosphatases [13,15].

Both HTS and in silico screening are widely used strategies for inhibitor discovery. Here, HTS identified 17 low-micromolar candidates, most of which share a common structural motif: an aromatic core with polar substituents. In silico approaches have also been applied to MPXV H1. One study screened FDA-approved and natural product libraries and identified mefloquine and aromadendrin as top candidates [23]. Notably, mefloquine was recently shown to inhibit MPXV in cell-based assays [24]. Inhibitors of variola virus H1 have also been reported [18], like our compounds, they feature aromatic scaffolds with polar groups, though they exhibited substantially lower potency in enzymatic assays [18]. Notably, the candidate compounds identified in our study are clinically applied or approved drugs. Referring to their well-established biosafety profiles, we found that these compounds, which have IC_50_ values in the nanomolar range against H1, can effectively inhibit H1 at clinically safe doses. These findings underscore the strength of our HTS-derived inhibitors.

Docking analysis revealed that the aromatic rings of compounds No. **41**, **47**, **57**, **109**, and **118** occupy the catalytic pocket, likely blocking substrate access. The critical residues mediating inhibitor interactions map to the P-loop (110–116) and WPD-loop (74–86), both of which are essential for catalysis [25,26]. The P-loop binds the phosphate moiety, whereas the WPD-loop stabilizes the thiolate form of Cys110, thereby enhancing nucleophilicity [26,27]. Beyond these motifs, Asn155 emerged as a key residue, forming hydrogen bonds or sulfur–nitrogen interactions with all five compounds analyzed. Interestingly, Asn155 also interacts with mefloquine and aromadendrin [23], suggesting it may serve as a conserved anchor point for inhibitor binding. Asp80 was likewise found to form hydrogen bonds with most compounds, except compound No. **47**, highlighting its role as an additional hotspot for ligand engagement. These findings point to Asn155 and Asp80 as particularly promising residues for structure-guided drug design.

Although we performed docking analysis for all candidate molecules, only five of them were successfully docked into the active site. These molecules have only one or two rings, whereas the larger molecules were unable to dock at this site. The relatively small size of the H1 active site (PDB: 8GZ4) may hinder direct docking of molecules containing more than two benzene rings. The candidates that were not successfully docked may inhibit H1 through one of the following mechanisms. First, some of them may bind at the active site through an induced-fit mechanism. Upon binding, they may induce conformational changes in regions surrounding the active site (for example, in the WPD-loop), a process not simulated in the docking analysis. It is acknowledged that docking analysis cannot perfectly simulate the bona fide interactions between protein and small molecules, as it is influenced by multiple factors, such as salt concentration in the buffer, pH, and temperature conditions. This is also reflected in that CDOCKER energy values and IC_50_ values may not be directly proportional, as the former is derived from docking simulations, while the latter is obtained from wet-lab experiments. Alternatively, some of the candidates may bind H1 at an allosteric site, leading to an overall conformational change in H1 and reduced activity. Experimental structures are needed to elucidate the location of the putative allosteric site and the interaction mode of such molecules.

In conclusion, we performed HTS against MPXV H1 and identified 17 potent inhibitors with favorable cytotoxicity profiles. Docking analyses clarified their inhibitory mechanisms and revealed their interactions with the WPD-loop, P-loop, and the N155 residue (Figure S4). Given that phosphatases are increasingly recognized as druggable targets [28,29], our results provide a strong foundation for the rational design and optimization of anti-poxvirus therapeutics targeting MPXV H1.

## Figures and Tables

**Table 1:** Top candidate compounds and the IC_50_ values.

Compound No.	Structures	Name	IC_50_ (nM)
**35**	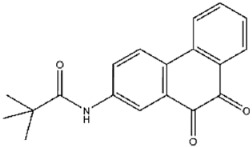	SF1670	154 ± 12
**41**	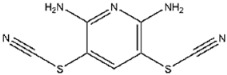	PR619	176 ± 7
**45**	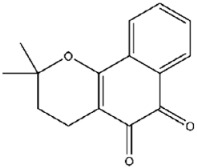	Beta-lapachone	348 ± 17
**47**	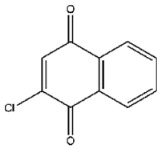	2-Chloro-1,4-naphthoquinone	837 ± 56
**57**	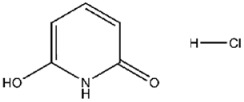	2,6-Dihydroxypyridine hydrochloride	911 ± 64
**59**	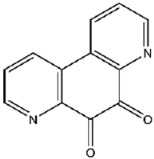	Phanquinone	241 ± 7
**68**	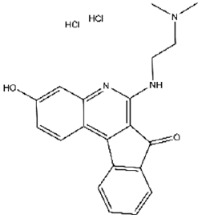	TAS-103 (dihydrochloride)	865 ± 16
**73**	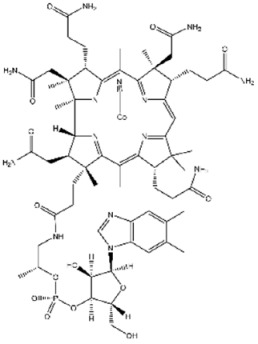	Vitamin B12	238 ± 5
**75**	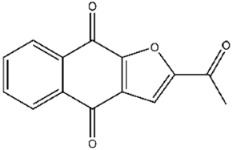	Napabucasin	957 ± 40
**79**	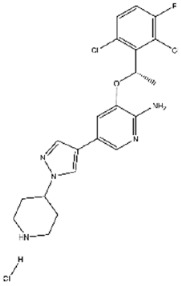	Crizotinib hydrochloride	946 ± 52
**88**	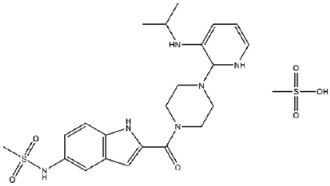	Delavirdine (mesylate)	987 ± 48
**89**	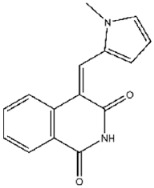	BYK204165	794 ± 46
**106**	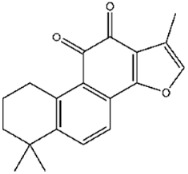	Tanshinone IIA	531 ± 19
**108**	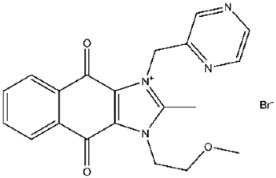	YM155	826 ± 65
**109**	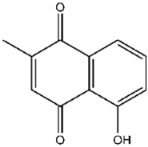	Plumbagin	874 ± 24
**111**	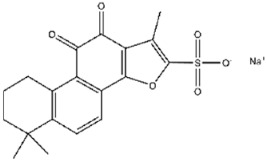	Tanshinone IIA sulfonate sodium	287 ± 10
**112**	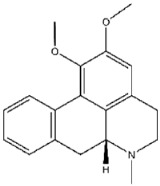	Nuciferine	752 ± 9
**118**	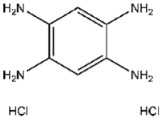	1,2,4,5-Benzenetetramine tetrahydrochloride	333 ± 30
**122**	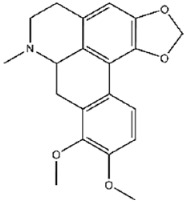	Crebanine	229 ± 21
**123**	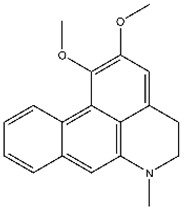	Dehydronuciferine	356 ± 25

**Table 2:** Information on CDOCKER results.

Compound No.	Name	CDOCKER Energy (kcal/mol)
**41**	PR619	−24.9
**47**	2-Chloro-1,4-naphthoquinone	−16.0
**57**	2,6-Dihydroxypyridine hydrochloride	−13.2
**109**	Plumbagin	−5.5
**118**	1,2,4,5-Benzenetetramine tetrahydrochloride	−22.5

## Data Availability

The original contributions presented in this study are included in the article/Supplementary Material. Further inquiries can be directed to the corresponding authors.

## Supplementary Materials

The following supporting information can be downloaded at https://www.mdpi.com/article/10.3390/v17111493/s1: Figure S1: The first round of IC_50_ determination; Figure S2: The second round of IC_50_ determination; Figure S3: Cell viability of potential inhibitors; Figure S4. The common motifs involved in candidate molecule binding.
