# Supplementary material for: High-Throughput Discovery of Inhibitors Targeting Monkeypox Virus H1 Phosphatase
Source: Viruses. 2025 Nov 12;17(11):1493. doi: 10.3390/v17111493 (PMC12656849; doi:10.3390/v17111493)
Supplement: Supplementary file 1 [file viruses-17-01493-s001.zip › Supplementary_Figure_caption.pdf]

Figure S1 The first round of IC_{50} determination.

The dephosphorylation activity of MPXV H1 was inhibited by the respective hit compounds, with their IC_{50} values indicated. All assays were performed in triplicate, and the results are presented as mean ± standard deviation.

Figure S2 The second round of IC_{50} determination.

The inhibition of MPXV H1 dephosphorylation activity by the respective hit compounds was re-evaluated after the initial IC_{50} screening, with updated IC_{50} values indicated. All assays were performed in triplicate, and the results are presented as mean ± standard deviation.

Figure S3. Cell viability of potential inhibitors.

Cell viability was determined by CCK-8 assay to evaluate the cytotoxicity of the compounds. The orange column indicates that the cell viability is >85%, and the red column indicates significant cytotoxicity. The experiment was performed in quadruplicate.

Figure S4. The common motifs involving in candidate molecule binding.

The overall view of MPXV H1 in complex with the candidate compounds is shown in the left panel, while a zoomed-in view of the binding site is shown in the right panel. The WPD-loop, P-loop, and N155 are the common motifs involved in interaction with the candidate molecules.
