# Supplementary figures and images for: High-Throughput Discovery of Inhibitors Targeting Monkeypox Virus H1 Phosphatase
Source: Viruses. 2025 Nov 12;17(11):1493. doi: 10.3390/v17111493 (PMC12656849; doi:10.3390/v17111493)

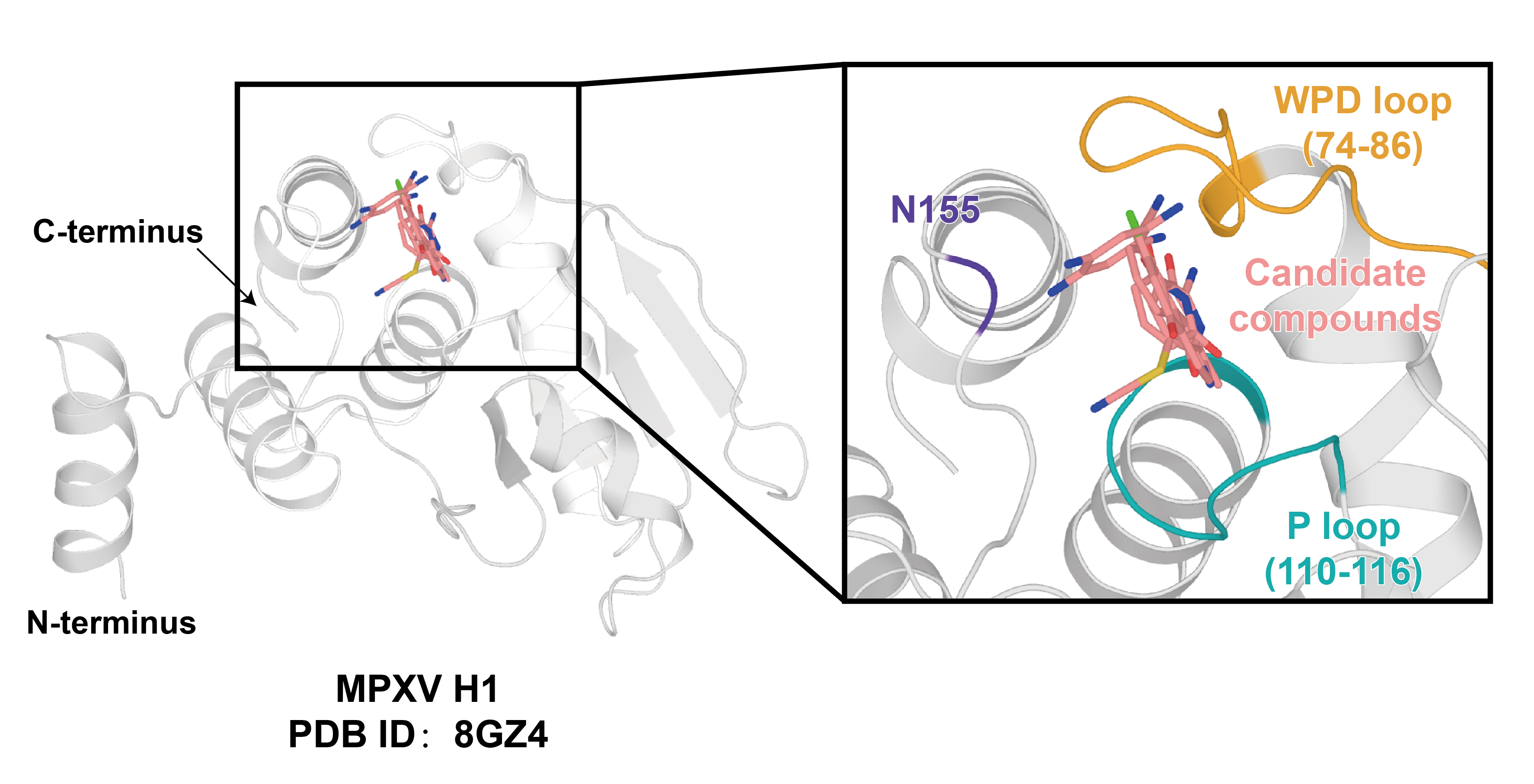

Supplement: Supplementary file 1 [file viruses-17-01493-s001.zip › Figure S4_revision.jpg]

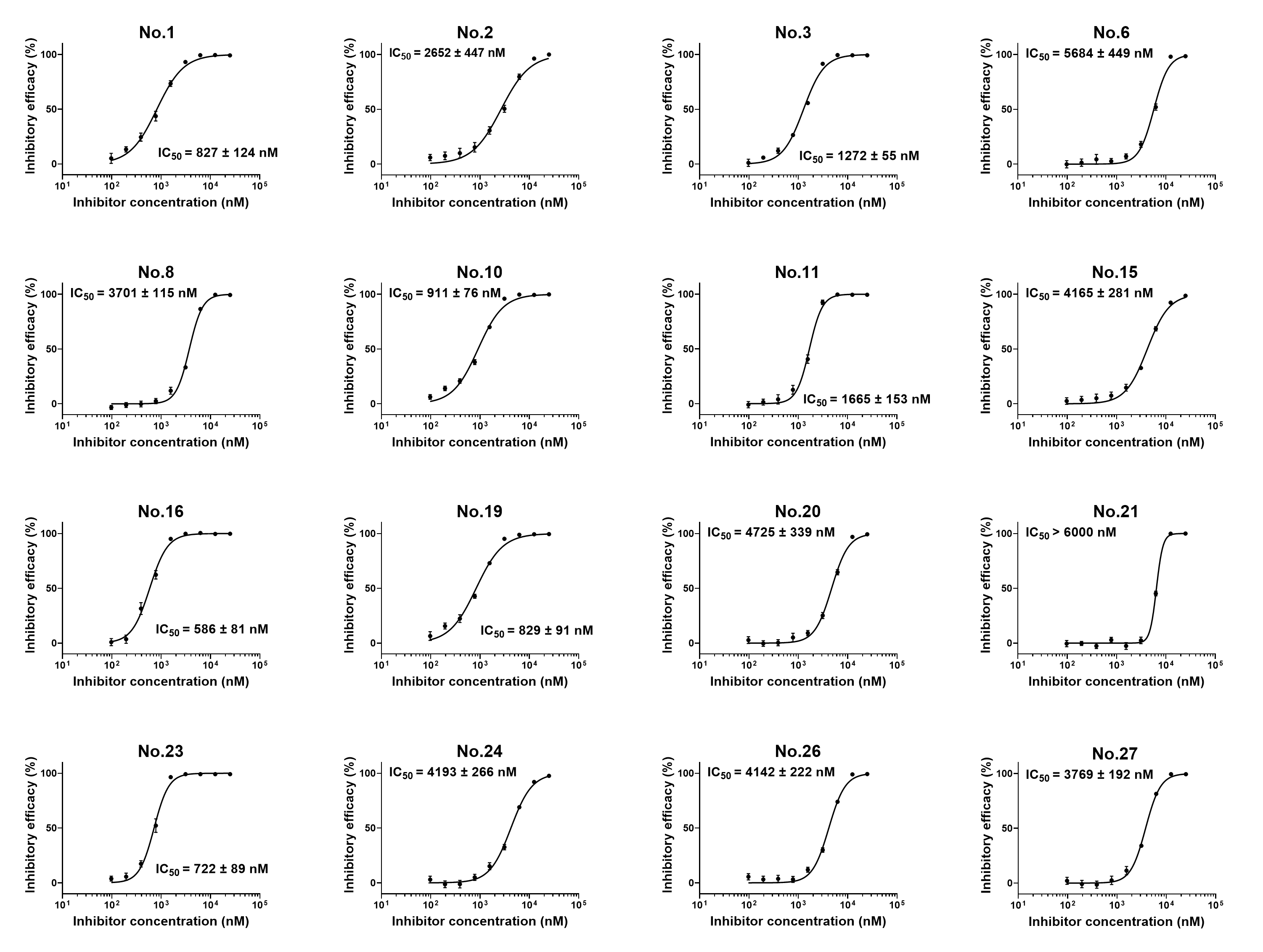

Supplement: Supplementary file 1 [file viruses-17-01493-s001.zip › Figure_S1_1.tif]

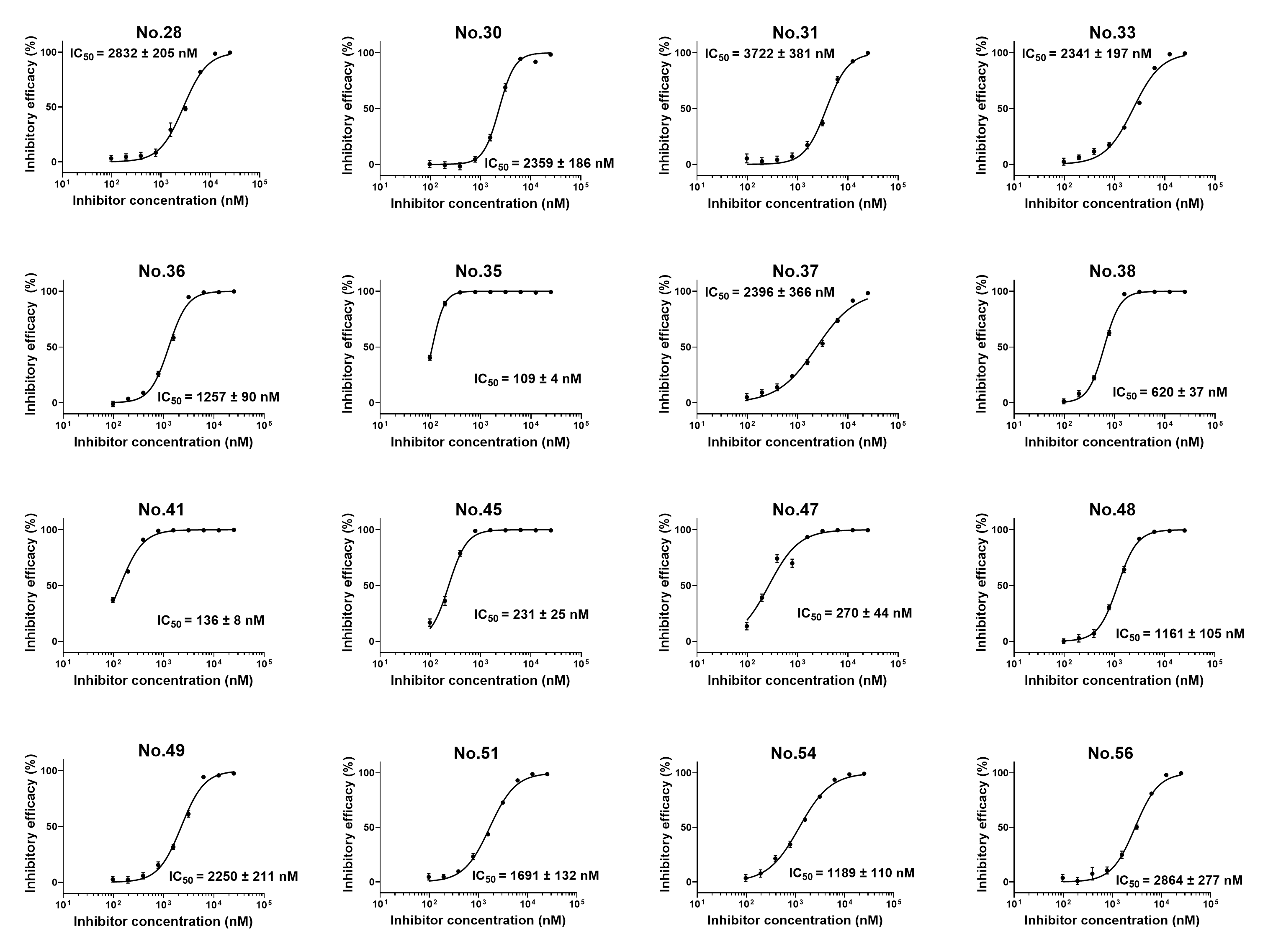

Supplement: Supplementary file 1 [file viruses-17-01493-s001.zip › Figure_S1_2.tif]

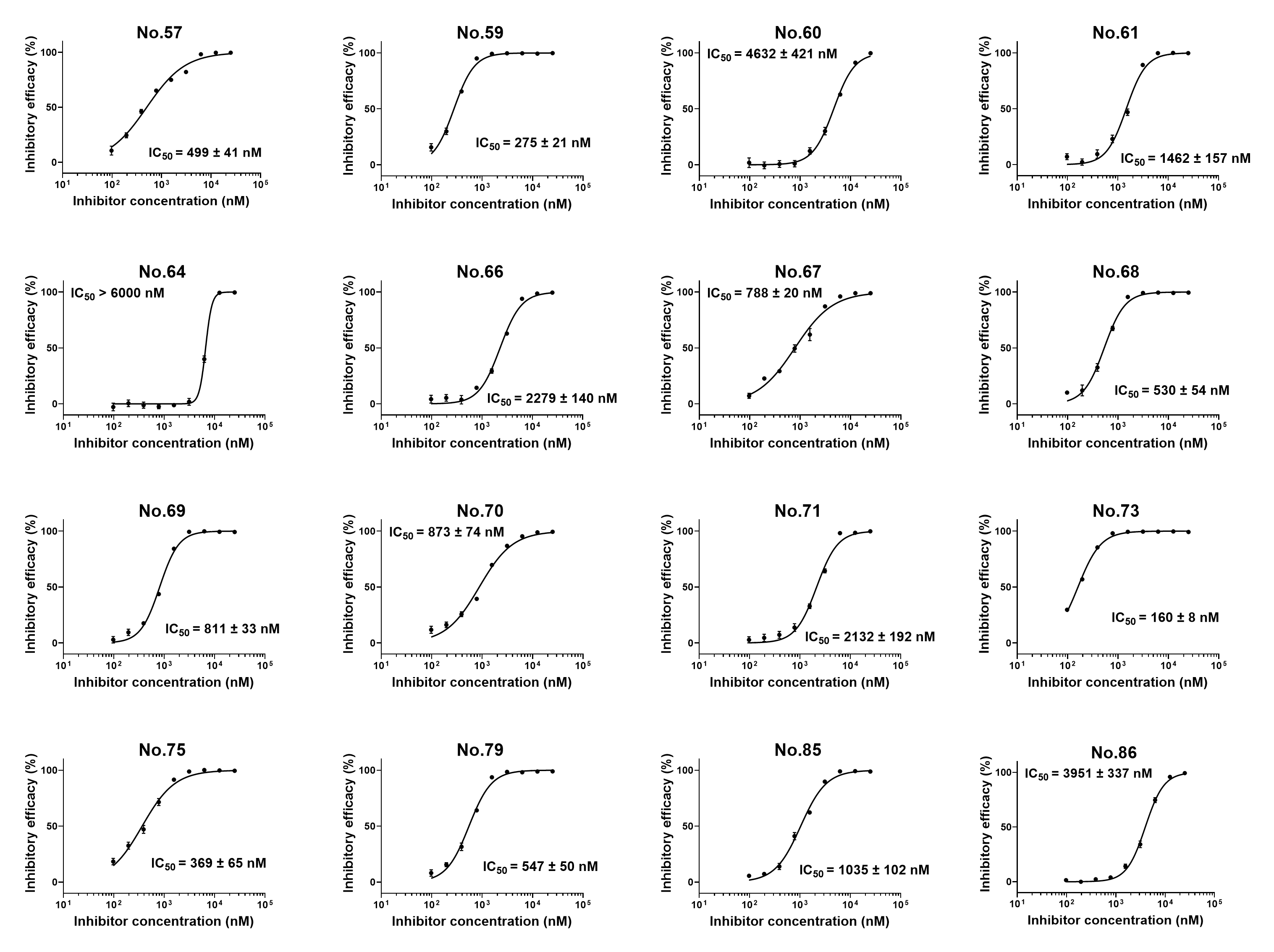

Supplement: Supplementary file 1 [file viruses-17-01493-s001.zip › Figure_S1_3.tif]

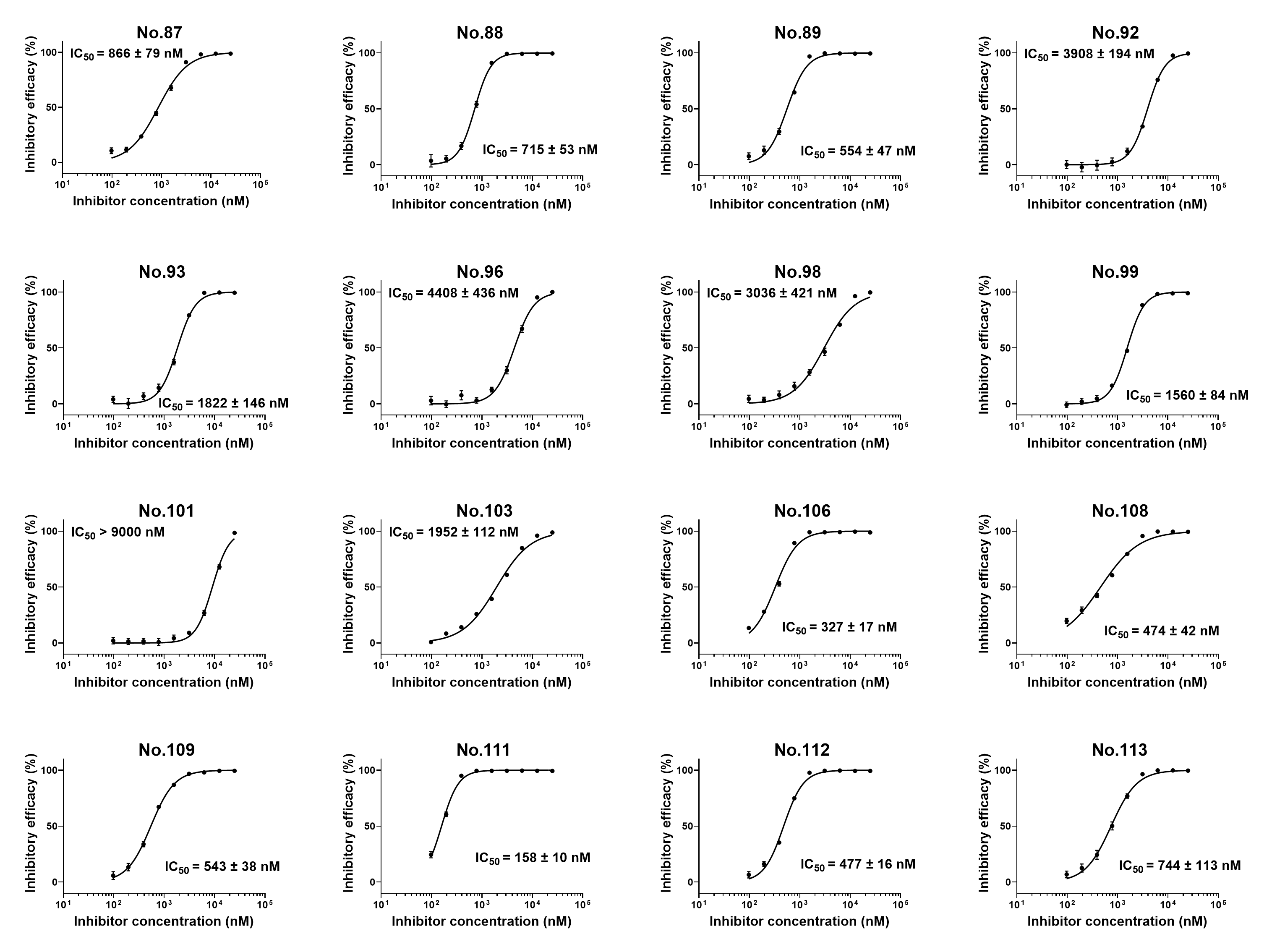

Supplement: Supplementary file 1 [file viruses-17-01493-s001.zip › Figure_S1_4.tif]

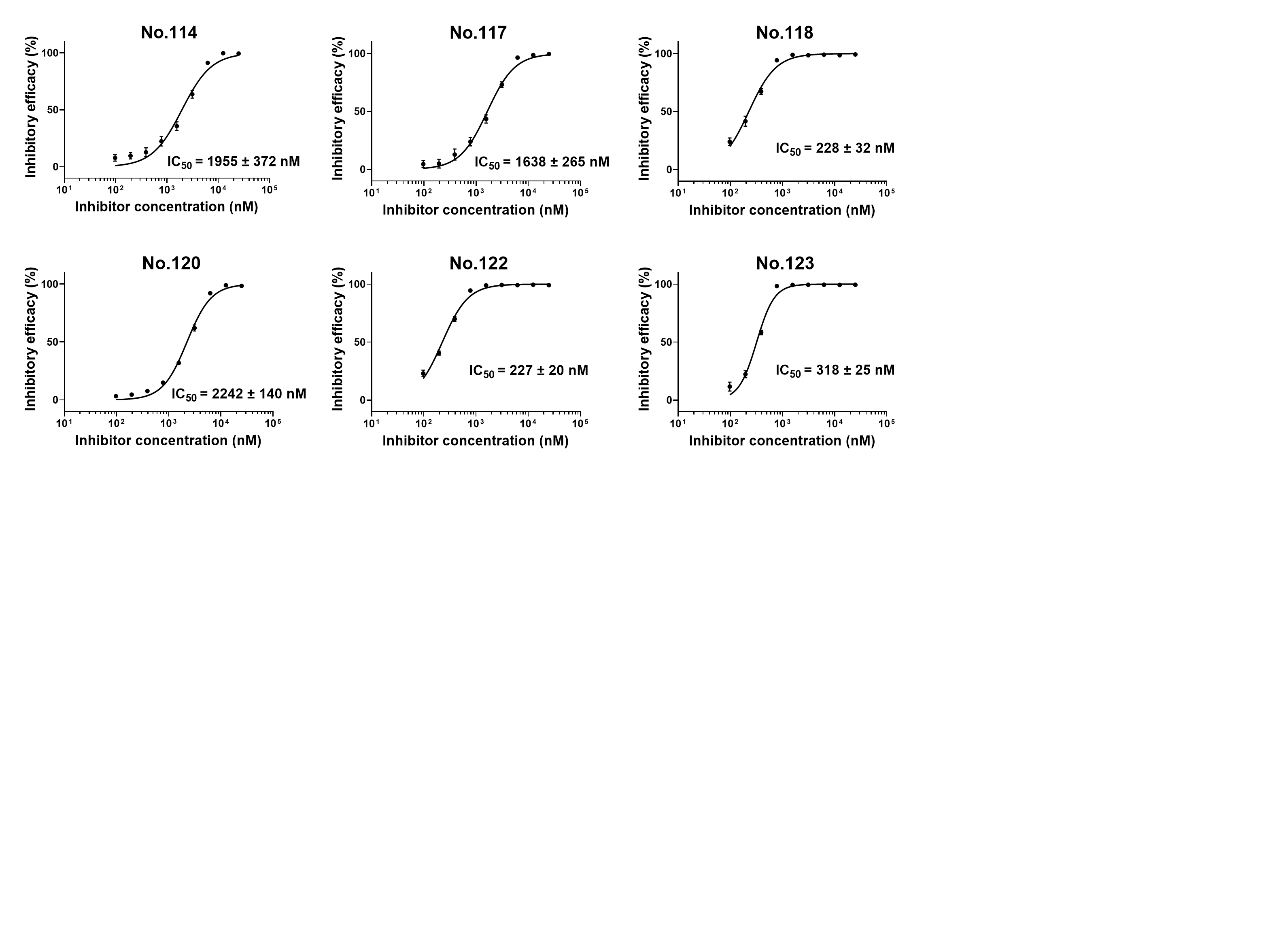

Supplement: Supplementary file 1 [file viruses-17-01493-s001.zip › Figure_S1_5.tif]

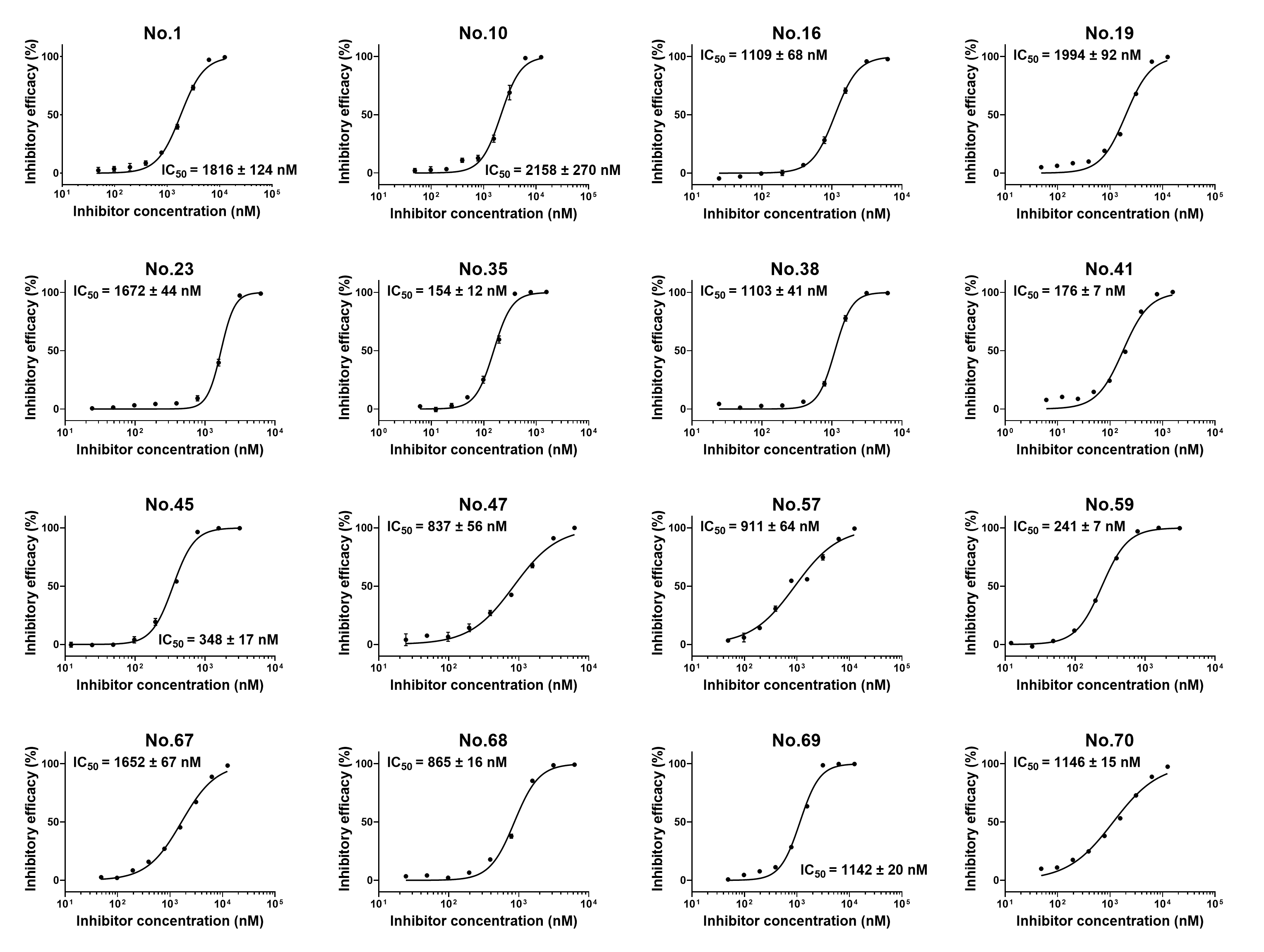

Supplement: Supplementary file 1 [file viruses-17-01493-s001.zip › Figure_S2_1.tif]

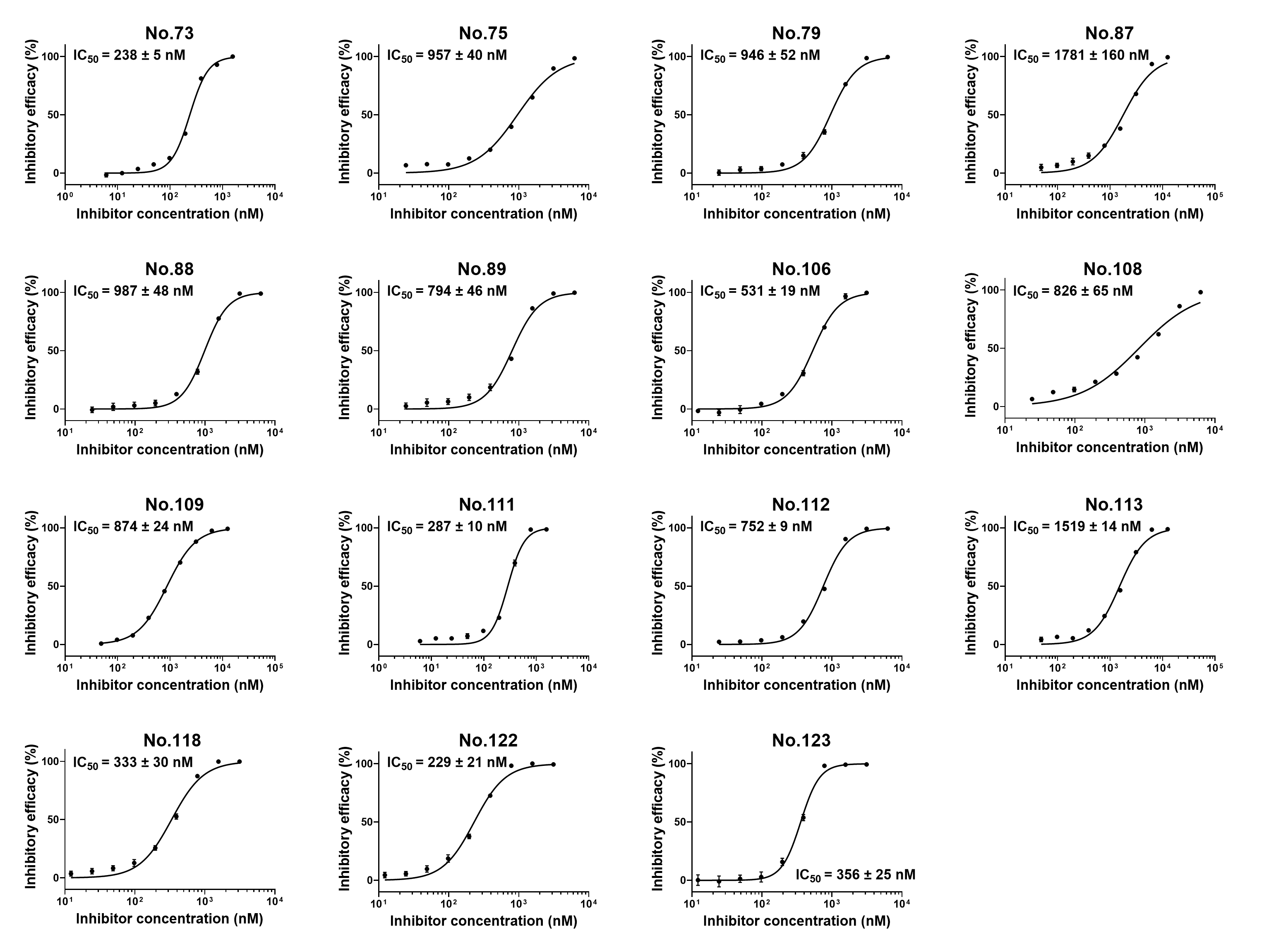

Supplement: Supplementary file 1 [file viruses-17-01493-s001.zip › Figure_S2_2.tif]

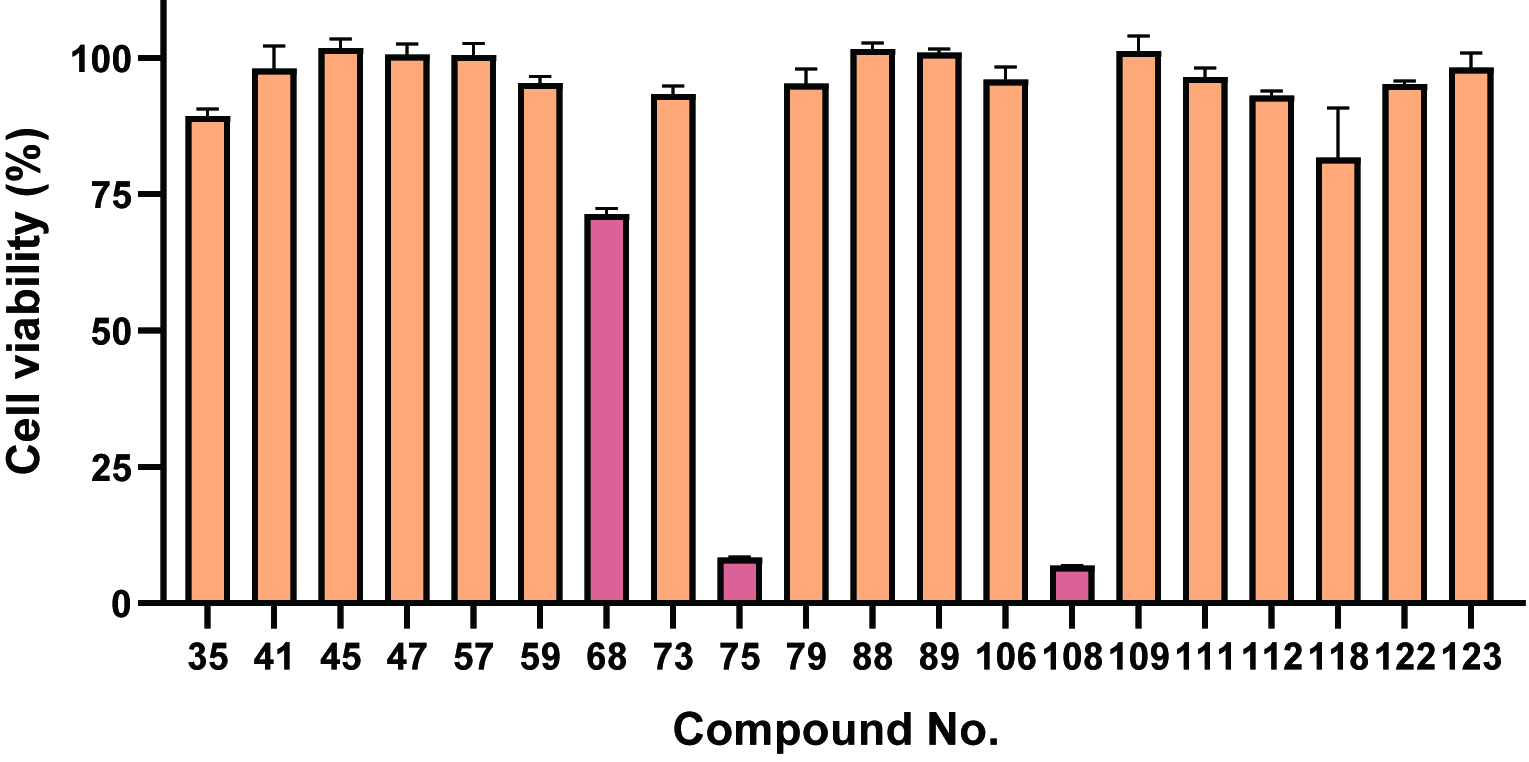

Supplement: Supplementary file 1 [file viruses-17-01493-s001.zip › Figure_S3.tif]
